# Bilateral Functional Tarsal Hyperextension Associated with Stifle Pathology Improved Following Stifle Stabilization in a Dog: A Case Report

**DOI:** 10.3390/vetsci13060518

**Published:** 2026-05-27

**Authors:** Woosung Jung, Hyeong-mok Kim, Su-jin Son, Hwi-yool Kim

**Affiliations:** 1 Laboratory of Veterinary Surgery, College of Veterinary Medicine, Konkuk University, 120 Neungdong-ro, Gwangjin-gu, Seoul 05029, Republic of Korea; hykim@konkuk.ac.kr; 2 EUM Animal Medical Center, 15 Dongtan-Daero 6-Gil, Hwaseong-si 18501, Republic of Korea; vetine@naver.com (H.-m.K.); dogazi0514@gmail.com (S.-j.S.)

**Keywords:** dog, cranial cruciate ligament, gait abnormality, medial patellar luxation, stifle, tarsal hyperextension

## Abstract

Abnormal tarsal posture in dogs may be associated with structural lesions such as common calcaneal tendon complex rupture, plantar-supporting structure injury, tarsocrural instability, or neurologic dysfunction; however, common calcaneal tendon rupture is typically associated with a plantigrade posture rather than the weight-bearing-dependent tarsal hyperextension pattern described in this case. A 3-year-old intact male Pomeranian developed bilateral weight-bearing-dependent tarsal hyperextension immediately after bilateral medial patellar luxation (MPL) surgery performed at another hospital. At our hospital, complete cranial cruciate ligament rupture of the right stifle and recurrent grade II MPL of the left stifle were identified. Right lateral fabellotibial suture stabilization using a LigaFiba^®^ 75 lateral suture and crimp system, with supportive reapplication of a 0.8 mm Kirschner wire at the previous tibial tuberosity transposition site, and left trochlear block recession with capsular resection and imbrication were performed. No direct surgical treatment of either tarsus was undertaken. During follow-up after stifle stabilization, bilateral tarsal hyperextension progressively decreased and was no longer clinically observed by 5 months. This case suggests that bilateral weight-bearing-dependent tarsal hyperextension may occur secondary to proximal stifle pathology even in the absence of major structural tarsal instability, and that hyperextension may resolve clinically following stifle stabilization.

## Simple Summary

Tarsal hyperextension in dogs is usually discussed in relation to structural failure of the tarsal-supporting apparatus, such as common calcaneal tendon or plantar-supporting structure injury, which may produce distal limb collapse or a plantigrade stance. This case describes a dog with bilateral functional weight-bearing-dependent tarsal hyperextension in which clinical examination did not identify major structural tarsal instability, but concurrent right cranial cruciate ligament rupture and recurrent left medial patellar luxation were present. During follow-up after stifle stabilization, the abnormal tarsal gait gradually improved and was no longer clinically observed, without direct treatment of either tarsus. The main clinical message is that when stance-phase tarsal hyperextension is observed without classic plantigrade collapse, evaluation should include proximal joints, especially the stifle, while also considering postoperative compensatory and neurologic factors.

## 1. Introduction

The canine tarsus plays a critical role in weight transfer and propulsion in the pelvic limb. During normal gait, dogs maintain a digitigrade posture, and the tarsal joint contributes to both weight bearing and propulsive force generation while remaining in a relatively flexed position [1]. Tarsal stability is maintained by the common calcaneal tendon complex, the plantar ligament, collateral ligaments, and surrounding periarticular soft tissues [2].

In dogs, structural disease of the tarsal-supporting apparatus has been described in association with common calcaneal tendon complex rupture, plantar-supporting structure injury, and tarsocrural instability. These conditions commonly involve distal limb collapse, calcaneal displacement, palpable or radiographic instability, or a plantigrade stance, and may require direct tarsal stabilization, reconstruction, external coaptation, or arthrodesis [2,3,4,5,6,7].

In contrast, stance-phase tarsal hyperextension may be observed during gait without classic plantigrade collapse or fixed passive tarsal instability. This distinction is important because a weight-bearing-dependent pattern may represent a functional or compensatory gait abnormality rather than primary structural failure of the tarsal-supporting apparatus. The pelvic limb functions as a kinetic chain in which the hip, stifle, and tarsal joints interact closely, and proximal joint pain or instability may alter weight distribution, propulsion, and distal limb posture during stance [1,8,9,10,11,12].

Reports describing a clinical association between stifle pathology and functional weight-bearing-dependent tarsal hyperextension are limited. We therefore describe a dog with bilateral, stance-phase-predominant tarsal hyperextension associated with concurrent stifle pathology, in which the gait abnormality gradually improved during follow-up after stifle stabilization without direct treatment of either tarsus. The case is presented to emphasize that evaluation of dogs with tarsal hyperextension should distinguish plantigrade collapse caused by structural tarsal failure from functional weight-bearing-dependent hyperextension patterns associated with the pelvic limb kinetic chain.

## 2. Case Description

### 2.1. History and Clinical Findings

A 3-year-old, 3.55 kg, intact male Pomeranian was referred for evaluation of gait abnormality. Three weeks before presentation, the dog had undergone bilateral corrective surgery for medial patellar luxation (MPL) at another hospital. The preoperative MPL grade and detailed surgical records from the referring hospital were not available. According to the owner, dragging of the right pelvic limb was observed immediately after the previous surgery. A fixation pin was removed at the referring hospital 10 days before presentation, but the abnormal pelvic limb gait persisted, and excessive extension of both tarsal joints during ambulation was noted thereafter.

**Figure 1:**
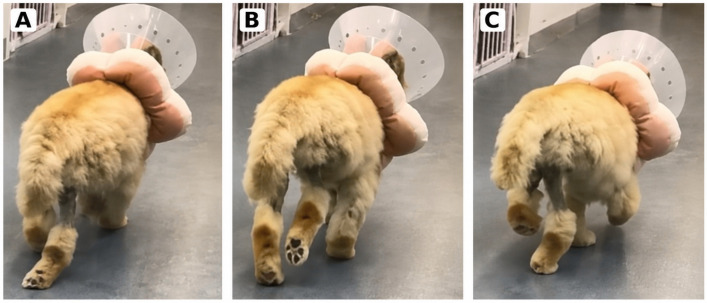
Preoperative gait. (**A**) The late stance phase showing excessive extension of the right tarsal joint during weight bearing. (**B**) The swing phase showing a relatively normal tarsal angle. (**C**) The weight-bearing stance demonstrating mild inversion of the right tarsal joint. The phase-dependent difference between stance and swing was considered clinically important because the abnormality was most apparent during weight bearing rather than being present as a fixed plantigrade posture. The preservation of a relatively normal tarsal angle during swing was considered supportive of a functional weight-bearing-dependent abnormality rather than a fixed plantigrade deformity.

On preoperative gait assessment, both pelvic limbs showed excessive tarsal extension during weight bearing, although a classic plantigrade stance was not observed (Figure 1). During the late stance phase, excessive extension of the tarsal joint was evident during weight bearing (Figure 1A), whereas a relatively normal tarsal angle was maintained during the swing phase (Figure 1B). Mild inversion of the right tarsal joint was also observed during weight bearing (Figure 1C).

Palpation did not reveal pain, swelling, or a palpable tendon defect in either tarsal region.

On passive range-of-motion assessment of the tarsal joints, the left side showed 170° of extension and 39° of flexion, whereas the right side showed 173° of extension and 37° of flexion. These findings did not suggest fixed passive hyperextension or gross tarsal instability.

Screening neurologic examination was unremarkable and did not identify spinal pain or overt proprioceptive deficits.

Orthopedic examination of the stifles revealed positive cranial drawer and tibial thrust in the right stifle, raising suspicion for cranial cruciate ligament rupture. Recurrent grade II medial patellar luxation was identified in the left stifle.

### 2.2. Radiographic Evaluation

Preoperative assessment included complete blood count, venous blood gas analysis, serum biochemistry, coagulation testing, proBNP testing, thoracic radiography, abdominal ultrasonography, electrocardiographic screening, and orthopedic radiography. The complete blood count, venous blood gas analysis, PT/aPTT, proBNP, and electrocardiographic screening were within reference limits. Serum biochemistry showed increased liver enzyme activities. Thoracic radiography did not identify a clinically relevant cardiopulmonary abnormality, although an undulating tracheal course was noted. Abdominal ultrasonography identified gallbladder sludge and mild hepatic parenchymal echogenicity change, considered most consistent with vacuolar hepatopathy.

**Figure 2:**
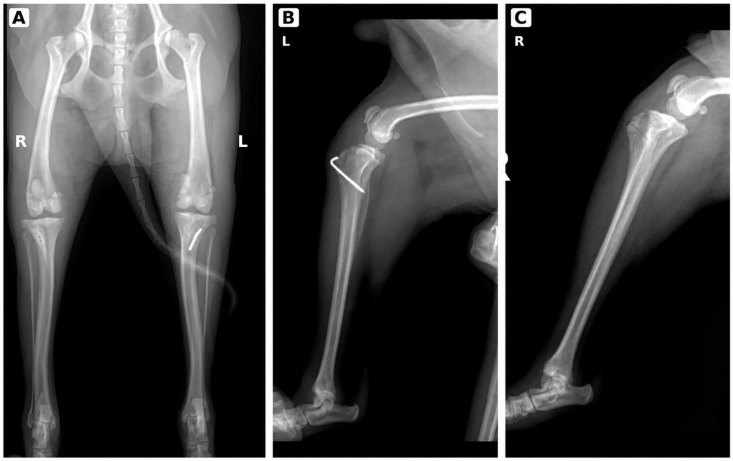
Preoperative radiographs. (**A**) Ventrodorsal pelvic projection including both stifles with right- and left-side markers. Pelvic limb alignment was assessed subjectively; no gross fracture, luxation, or severe osseous malalignment was identified, although this projection is susceptible to positional variation. (**B**) A left stifle lateral radiograph showing a previously placed tibial tuberosity fixation wire from prior medial patellar luxation surgery. (**C**) A right stifle lateral radiograph obtained for assessment of suspected stifle instability. The radiograph alone was not considered diagnostic for cranial cruciate ligament rupture and was interpreted in conjunction with orthopedic examination findings. L and R indicate the left and right sides, respectively.

Orthopedic radiographs were obtained using a digital radiography system (LOVET-120, SynicsRay, Seoul, Republic of Korea). The exposure settings were 50 kVp, 140 mA, and 4.94 mAs for the ventrodorsal pelvic projection including both stifles, and 45 kVp, 140 mA, and 4.94 mAs for the lateral stifle projections (Figure 2).

A ventrodorsal pelvic radiograph including both stifles showed no gross fracture, luxation, or severe osseous malalignment within the field of view. Pelvic limb alignment was assessed subjectively, and no objective angular measurements were performed.

The left stifle lateral radiograph showed a previously placed tibial tuberosity fixation wire associated with prior MPL surgery (Figure 2B).

**Figure 3:**
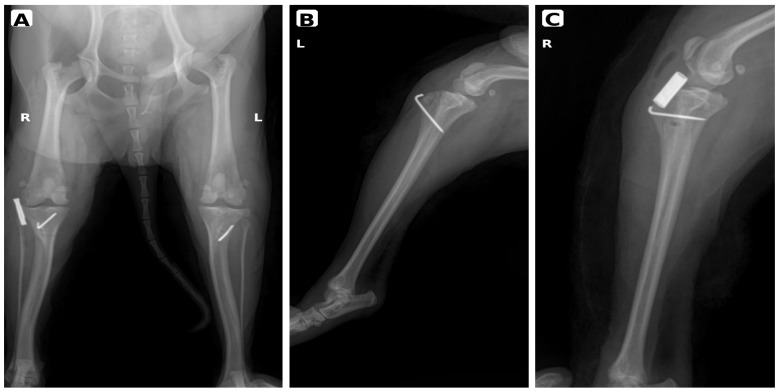
Immediate postoperative radiographs. (**A**) Ventrodorsal pelvic projection with right- and left-side markers showing bilateral postoperative implant placement and immediate postoperative alignment. (**B**) A left stifle lateral radiograph showing fixation at the previous tibial tuberosity transposition site after revision medial patellar luxation surgery. (**C**) A right stifle lateral radiograph showing the extracapsular stabilization construct and supportive reapplication of fixation at the previous tibial tuberosity transposition site. These radiographs were obtained to document implant position and immediate postoperative alignment rather than to assess long-term healing. L and R indicate the left and right sides, respectively.

The right stifle lateral radiograph did not independently confirm cranial cruciate ligament rupture. However, when interpreted together with the positive cranial drawer and tibial thrust identified on orthopedic examination, the findings were considered clinically consistent with right stifle instability (Figure 2C). Immediate postoperative radiographs were obtained to document implant placement and immediate postoperative alignment; no immediate postoperative osseous complication was identified (Figure 3).

### 2.3. Surgical Intervention

For preanesthetic medication, cefazolin (20 mg/kg IV), famotidine (0.5 mg/kg IV), and butorphanol (0.1 mg/kg IV) were administered. Anesthesia was induced with propofol (4 mg/kg IV), and after endotracheal intubation, anesthesia was maintained with isoflurane in oxygen. Hartmann’s solution was administered at 18 mL/h during anesthesia. The total anesthesia and surgical times were approximately 2 h each. Bradycardia was noted during anesthesia, but recovery was uneventful.

The dog was positioned in dorsal recumbency, and both pelvic limbs were prepared aseptically and draped in a standard orthopedic fashion. After induction of anesthesia and before surgical stabilization, orthopedic examination was repeated. Right stifle instability was reassessed, and a positive tibial thrust test was again confirmed. Both tarsal joints were palpated and manually examined under anesthesia before stifle surgery; clinical examination did not identify gross tarsal instability, palpable tendon defects, or findings suggestive of major structural failure of the tarsal-supporting apparatus.

In the right stifle, a limited parapatellar arthrotomy was performed. The infrapatellar fat pad was partially retracted and trimmed as needed to improve visualization of the intercondylar region. A small amount of viscous, pink-tinged synovial fluid was observed. Complete rupture of the cranial cruciate ligament was identified intraoperatively, and residual ruptured cranial cruciate ligament fibers were carefully removed. The menisci were inspected and probed to the extent possible, although complete systematic meniscal evaluation was not performed. Extracapsular stabilization was then performed using a lateral fabellotibial suture technique with a LigaFiba^®^ 75 lateral suture and crimp system (Veterinary Instrumentation, Sheffield, UK). The suture was passed around the lateral fabella and through a bone tunnel in the proximal tibia, tensioned with the stifle held in functional alignment, and secured using a crimp. Because the previous tibial tuberosity transposition had been performed only 3 weeks before referral and the fixation pin had been removed 10 days before presentation, a 0.8 mm Kirschner wire was reapplied to the previous tibial tuberosity transposition site to provide additional support during the early healing period.

In the left stifle, recurrent grade II medial patellar luxation was confirmed. The previous tibial tuberosity transposition alignment was considered adequate; therefore, additional tibial tuberosity transposition was not performed. Mild superficial cartilage abrasion was observed on the medial articular surface of the patella. The trochlear groove was re-exposed, and trochlear block recession was performed by elevating the osteochondral trochlear block, removing additional underlying bone, and replacing the block in a recessed position to improve trochlear depth and patellar engagement. Redundant and lax capsular tissue, particularly on the lateral aspect, was resected and imbricated to improve patellar stability. Patellar tracking was reassessed through passive flexion and extension of the stifle.

The long digital extensor tendons were grossly normal bilaterally. No direct surgical treatment or external coaptation was applied to either tarsus.

The surgical sites were closed routinely in layers. Postoperative medication during hospitalization included cefazolin (20 mg/kg IV BID), famotidine (0.5 mg/kg IV BID), meloxicam (0.1 mg/kg IV SID), and butorphanol (0.15 mg/kg IV q4h).

### 2.4. Outcome

Postoperative gait was assessed by serial clinical examination and visual gait observation. Video documentation was available only at selected follow-up time points and was not obtained at every postoperative assessment. Follow-up goniometric measurements and quantitative dynamic tarsal angle analysis were not performed. At postoperative day 2 (POD2), gait assessment showed persistent tarsal hyperextension on the right side, whereas the left side showed mild improvement compared with the preoperative state. At 2 weeks after surgery, right-sided tarsal hyperextension had decreased, and the left pelvic limb appeared nearly clinically normal on visual gait assessment; overall gait function had improved.

**Figure 4:**
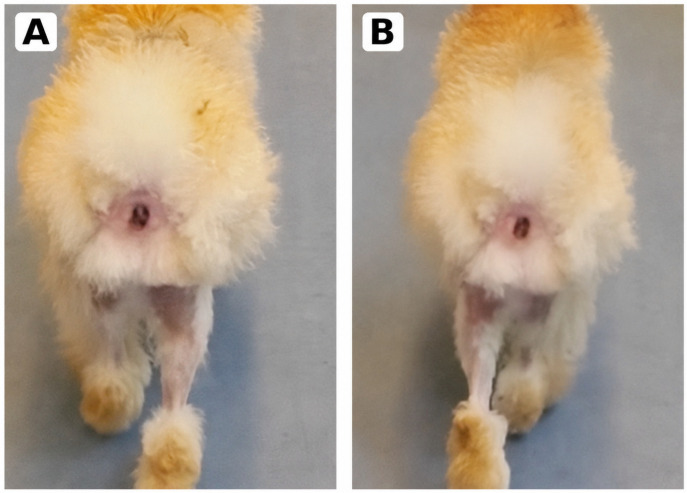
Postoperative gait at 4 weeks. Mild residual right-sided tarsal hyperextension was still observed during weight bearing, but overall pelvic limb alignment had improved compared with the preoperative gait. (**A**) A single-limb stance showing residual, stance-phase-dependent right tarsal hyperextension. (**B**) A bilateral stance showing improved weight-bearing alignment. These images illustrate partial clinical improvement in the functional weight-bearing-dependent tarsal hyperextension pattern without direct treatment of either tarsus.

At 4 weeks after surgery, mild residual tarsal hyperextension remained on the right side, whereas the left pelvic limb appeared clinically normal on visual gait assessment, and overall weight-bearing alignment had improved (Figure 4). At 10 weeks after surgery, right-sided tarsal hyperextension was observed only intermittently, while the left pelvic limb remained clinically normal on visual assessment.

At the final follow-up examination, 5 months after surgery, bilateral tarsal hyperextension was no longer clinically observed, and the gait was considered clinically normal on visual assessment. The abnormal gait pattern improved gradually without any direct intervention on the tarsal joints.

## 3. Discussion

The most notable feature of this case was bilateral weight-bearing-dependent tarsal hyperextension without clinical evidence of major structural tarsal instability, followed by gradual clinical improvement during follow-up after stabilization of concurrent stifle lesions without direct treatment of either tarsus.

Most canine tarsal instability reports describe structural failure of the tarsal-supporting apparatus, including common calcaneal tendon complex rupture, plantar-supporting structure injury, or tarsocrural instability. These lesions typically result in calcaneal displacement, hock collapse, palpable instability, or a plantigrade stance, and many cases require surgical reconstruction, external coaptation, or arthrodesis [2,3,4,5,6,7]. The present dog differed from these structural presentations: a classic plantigrade stance was not observed, excessive tarsal extension was most apparent during weight bearing, and a relatively normal tarsal angle was maintained during the swing phase. This phase-dependent pattern supported interpretation as functional weight-bearing-dependent hyperextension rather than fixed plantigrade collapse caused by major structural failure of the tarsal-supporting apparatus.

The interpretation of functional hyperextension was based on the combined clinical pattern rather than on definitive exclusion of all possible tarsal disease. Clinically important common calcaneal tendon rupture was considered less likely because the dog did not show a classic plantigrade stance, distal calcaneal displacement, a palpable tendon defect, marked tarsal pain or swelling, or persistent abnormal tarsal posture during the swing phase. Plantar-supporting structure injury was also considered less likely because passive tarsal assessment and manual examination under anesthesia did not identify fixed passive hyperextension or gross tarsal instability. In addition, the abnormality was most evident during weight bearing, whereas a relatively normal tarsal angle was maintained during the swing phase. This phase-dependent pattern supported the interpretation of a functional, load-dependent abnormality rather than passive structural collapse of the tarsal-supporting apparatus. Nevertheless, subtle tarsal soft-tissue injury could not be definitively excluded because ultrasonography, computed tomography, or magnetic resonance imaging of the tarsal region was not performed.

The pelvic limb functions as a kinetic chain in which the hip, stifle, and tarsal joints interact during weight transfer and propulsion [1,8,9,10,11,12]. Stifle disorders, including cranial cruciate ligament rupture and medial patellar luxation, can alter pelvic limb biomechanics, stance-phase stability, and adjacent joint motion [8,9,10,11,12]. In dogs with cranial cruciate ligament insufficiency, abnormal stifle kinematics are most pronounced during stance, and changes in hip and tarsal motion have been described as compensatory responses to abnormal stifle function [10,11,12]. In dogs with medial patellar luxation, reduced stifle range of motion may be accompanied by compensatory increases in hip and tarsal active range of motion, and these adaptations may diminish as stifle function improves after surgical correction [9]. Although the present case involved recurrent grade II medial patellar luxation and contralateral cranial cruciate ligament rupture rather than isolated grade III medial patellar luxation, these findings support the broader concept that stifle dysfunction can be associated with compensatory changes in adjacent hip and tarsal joint motion.

In addition to altered distal biomechanics secondary to proximal instability, compensatory pain avoidance mechanisms may have contributed to the observed gait pattern. Hindlimb lameness in dogs can result in unloading of the affected pelvic limb, redistribution of vertical ground reaction forces, and displacement of the body center of mass [13]. Pain or discomfort associated with the stifle pathology may therefore have contributed to partial pelvic limb unloading and a forward and/or asymmetric shift in body-weight distribution during stance. Such altered weight distribution may reduce normal pelvic limb propulsion and require compensatory recruitment of the gastrocnemius-Achilles mechanism during late stance. Because the gastrocnemius contributes to tarsal extension and active push-off during stance, compensatory recruitment or altered timing of this mechanism may have accentuated tarsal extension during weight bearing [14]. This interpretation is consistent with the observation that excessive tarsal extension was most apparent during the stance phase, whereas a relatively normal tarsal angle was maintained during the swing phase.

Abnormal tarsal extension can also be associated with neurologic disease. In particular, dysfunction of the sciatic or tibial nerve may alter the balance of muscles controlling tarsal extension and flexion and thereby produce abnormal gait patterns [15]. In the present dog, the screening neurologic examination did not identify spinal pain, proprioceptive deficits, or progressive neurologic deterioration, and the gait abnormality gradually improved during follow-up after stifle stabilization. These findings made a primary neurologic disorder less likely; however, subtle neurologic dysfunction was not definitively excluded because advanced neurologic evaluation, electrodiagnostic testing, and imaging of the sciatic or tibial nerve were not performed.

In this case, right cranial cruciate ligament rupture and recurrent left medial patellar luxation may each have influenced pelvic limb load transfer and propulsion in different ways. The abnormality was therefore interpreted as clinically functional rather than as hyperextension caused by fixed structural rupture of the tarsal-supporting apparatus. However, this interpretation should be understood as a clinically meaningful temporal association rather than proof of a direct causal relationship.

The history of previous bilateral medial patellar luxation surgery is an important confounding factor. The abnormal gait was first noticed immediately after the previous surgery, and a fixation pin was removed before referral. Therefore, previous surgery-related factors, implant-associated discomfort, altered postoperative limb use, or the recovery process may have contributed to the abnormal stance mechanics. The gradual improvement observed during follow-up after stabilization of the concurrent stifle lesions without direct tarsal treatment supports an association, but a direct causal relationship between the stifle lesions identified at our hospital and the tarsal hyperextension cannot be established from this single case.

These findings indicate that evaluation of dogs with tarsal hyperextension should not be limited to the tarsal joint itself. Especially when a classic plantigrade stance or clear structural tarsal lesion is absent, proximal joint instability, postoperative compensatory mechanisms, pain-related unloading, and neurologic causes should all be considered in the differential assessment.

This report has several limitations. First, postoperative improvement was assessed by serial clinical examination and visual gait observation rather than by quantitative gait analysis, force-plate analysis, follow-up goniometric measurements, or dynamic tarsal angle analysis. Video documentation was available only at selected follow-up time points and was not obtained consistently at every postoperative visit.

Second, structural and imaging assessment was limited. No follow-up radiographs were obtained beyond the immediate postoperative period, and pelvic limb alignment was assessed subjectively on the available radiographs without objective angular measurements. Advanced imaging of the tarsal soft tissues, including ultrasonography, computed tomography, or magnetic resonance imaging, was not performed; therefore, subtle injury of the common calcaneal tendon complex, plantar ligament, or other periarticular soft tissues could not be definitively excluded.

Third, complete systematic meniscal evaluation, synovial fluid analysis, advanced neurologic evaluation, electrodiagnostic testing, and diagnostic imaging of the sciatic or tibial nerve were not performed. In addition, details of the previous procedure and immediate postoperative records from the referring hospital were limited. Accordingly, this case supports a clinically meaningful association rather than proof of causality. Despite these limitations, it provides a clinically relevant example showing that bilateral tarsal hyperextension was no longer clinically observed during follow-up after stabilization of concurrent stifle pathology without direct treatment of the tarsus.

## 4. Conclusions

This case suggests that bilateral, weight-bearing-dependent tarsal hyperextension may occur in clinically meaningful temporal association with concurrent stifle pathology and may no longer be clinically evident on visual gait assessment during follow-up after stifle stabilization, even without direct treatment of the tarsus. In dogs with stance-phase tarsal hyperextension and no clinical evidence of major structural tarsal instability or classic plantigrade collapse, the diagnostic work-up should include assessment of proximal joints, particularly the stifle, while also considering postoperative compensatory and neurologic factors.

## Data Availability

The original contributions presented in this study are included in the article and Supplementary Materials. Further inquiries can be directed to the corresponding author.

## Supplementary Materials

The following supporting information can be downloaded at: https://www.mdpi.com/article/10.3390/vetsci13060518/s1, Video S1: Raw video of Figure 1; Video S2: Raw video of Figure 4.
